# Real-time monitoring of *Arundo donax* response to saline stress through the application of in vivo sensing technology

**DOI:** 10.1038/s41598-021-97872-6

**Published:** 2021-09-20

**Authors:** Janni Michela, Cocozza Claudia, Brilli Federico, Pignattelli Sara, Vurro Filippo, Coppede Nicola, Bettelli Manuele, Calestani Davide, Francesco Loreto, Andrea Zappettini

**Affiliations:** 1 National Research Council of Italy, Institute of Materials for Electronics and Magnetism (IMEM), National Research Council (CNR), Parco Area delle Scienze 37/A, 43124 Parma, Italy; 2 National Research Council of Italy, Institute of Bioscience and Bioresources (IBBR), National Research Council (CNR), Via Amendola 165/A, 70126 Bari, Italy; 3 Department of Agriculture, Food, Environment and Forestry (DAGRI), University of Florence, via San Bonaventura 13, 50145 Florence, Italy; 4 National Research Council of Italy, Institute for the Sustainable Plant Protection (CNR - IPSP), Via Madonna del Piano 10, 50019 Sesto Fiorentino, Italy; 5 National Research Council of Italy – Department of Biology, Agriculture and Food Sciences, (CNR-DISBA), P. Le Aldo Moro, 00185 Roma, Italy; 6 Department of Biology, University of Naples Federico II, Naples, Italy; 7 Laboratory of Environmental and Life Sciences, University of Nova Gorica, Vipavska cesta 13, 5000 Rožna Dolina, Nova Gorica, Slovenia

**Keywords:** Sensors and biosensors, Plant stress responses, Plant sciences, Materials science

## Abstract

One of the main impacts of climate change on agriculture production is the dramatic increase of saline (Na^+^) content in substrate, that will impair crop performance and productivity. Here we demonstrate how the application of smart technologies such as an in vivo sensor, termed bioristor, allows to continuously monitor in real-time the dynamic changes of ion concentration in the sap of *Arundo donax* L. (common name giant reed or giant cane), when exposed to a progressive salinity stress. Data collected in vivo by bioristor sensors inserted at two different heights into *A. donax* stems enabled us to detect the early phases of stress response upon increasing salinity. Indeed, the continuous time-series of data recorded by the bioristor returned a specific signal which correlated with Na^+^ content in leaves of Na-stressed plants, opening a new perspective for its application as a tool for in vivo plant phenotyping and selection of genotypes more suitable for the exploitation of saline soils.

## Introduction

Soil salinity is a recurrent and adverse environmental stressor that impairs crop growth and productivity, and negatively affects food security^1,2^. The area of saline soil is as large as 397 million ha^3^. It is well established that a high soil salinity impacts on the survival of many plant species, especially halophytes^4^. To address lands recovery from salinity by promoting alternative land uses, renewable alternative energy sources is steadily rising, thus plant species (genotypes) resistant to salinity must be identified^5^. Salinity may be tolerated by plants through different strategies involving either ‘tissue tolerance’, where toxic ions are compartmentalized into specific tissues, cells and subcellular organelles, or ‘toxic ion exclusion’, where plant survival is maintained by fast translocation of Na^+^ out of the plant to avoid excessive accumulation into the shoot apical meristems^6–8^.

So far, a wide range of physiological, biochemical and molecular analyses have been employed to detect and study salinity stress and how it adversely affects plant growth and performance^2,9–14^. However, these methods, rely on destructive assays, on indirect measurement or on digital imaging, and thus only allow indirect access to the effects of saline stress on plants performances^8,15–19^. The electrical impedance spectroscopy (EIS) is proposed to study the behavior and properties of cell membranes in plants to investigate the plant tolerance to different abiotic stress, such as drought^20–23^and salt stress^24^. Because of the passive electrical properties, plant tissue impedance is related to cellular ionic content, membrane structures and viscosity^23^ and is determined by the observation of the tissue electrical response through two external electrodes placed on both side of the sample leaves^24,25^.

The availability of a novel technology able to monitor continuously and in vivo plant responses to environmental constraints, and to be applicable in open field, would allow us to early identify and follow the onset of mechanisms triggered within plants by the occurrence of salinity stress. Recently, a novel sensor has been developed to continuously and in in vivo monitor variations of the plant sap ion composition and concentration^26^. This device, named ‘bioristor’, is an organic electrochemical transistor (OECTs) realized on a textile fiber and integrated into the plant stem. An organic electrochemical transistor (OECT) is made of a conducting polymer film in contact with an electrolyte and a gate electrode immersed in it^27^. The working principle of an OECT is based on doping-state changes in the semiconductor channel material due to electrolyte-ion injections which modify the electrical conductivity^28^.

A positive input voltage at the gate electrode modulates the channel current by pushing cations from the electrolyte into the PEDOT: PSS matrix modifying the channel conductivity^29^. The bioristor was proven to be effective in determining the cation (i.e. Na^+^, K^+^, Ca^2+^, Mg^2+^) concentration of water and other organic liquids^30–33^ in the range of 10^–1^-10^–4^ M with the limitation of not being selective for a single cation^30–33^. In addition, the pioneering application of bioristor in dicotyledonous plant species, such as tomato, successfully allowed detection of changes occurring in the plant sap composition following the day/night circadian cycle^26^, as well as early detection of plant stress conditions under drought^33^. The correlations among bioristor sensor response, changes in relative humidity, and vapour pressure deficit have been recently demonstrated, proposing the bioristor as a novel sensor to improve water use efficiency^34^.

**Figure 1:**
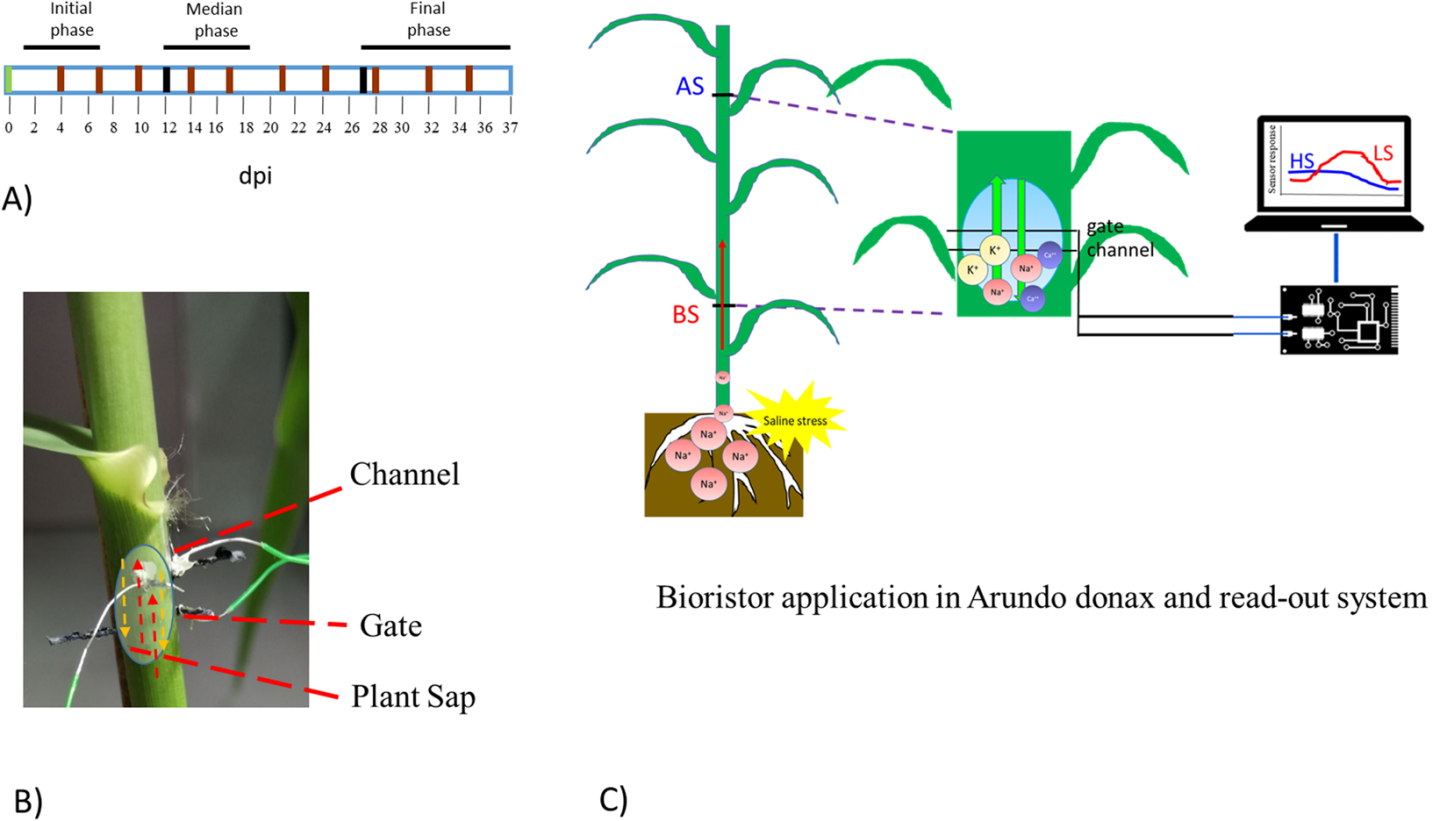
Real-time monitoring of salinity response in *A. donax* with bioristor. (**A**) Experimental set-up: the green block indicates the insertion of sensors into *A. donax* stem; brown lines indicate saline treatments (200 mM NaCl) and the black lines correspond to the sensor maintenance. Destructive measurements were performed in the initial phase at 1 dpi, in the median phase at 18 dpi and in the finale phase at 24 dpi; (**B**) A detailed view of a biostor inserted into *A. donax* stem. Dashed yellow and red lines represent the sap movement directions. (**C**) Scheme of the bioristor installation and function principle into *A. donax* plants; two sensors were inserted: one between the 2nd and the 3rd leaf (apical sensor, AS), and a second one between the 5th and 6th leaf (basal sensor, BS).

Here, we report on the application of the bioristor to monitor *Arundo donax* plants challenged with a severe salt stress (Fig. 1). *A. donax* is a perennial monocot species responsive to salt treatment^14^, and a promising crop for both bioenergy and biomass feedstock^35^. In companion studies, *A. donax* showed ability to cope salinity coupled with high P concentration by revealing efficient stomatal regulation^14^ and by enhancing biosynthesis of antioxidants^26^. In companion studies, *A. donax* showed ability to grown in saline soil^14^ by revealing efficient stomatal regulation^14^ and by enhancing biosynthesis of antioxidants^26^. Indeed, *A. donax* shows the spread in altered stream hydrology or sea-level rise, namely fields dominated by saline soils^10^. In particular, the objectives of this work were: (1) to investigate the bioristor sensitivity to the overall salinity which is mostly controlled by the Na concentration in the plant sap; (2) to evaluate whether the bioristor can be used as a rapid and non-destructive method for early detection of salt stress; (3) to increase knowledge of the mechanisms employed by *A. donax* to withstand saline stress.

## Results

In this study, the bioristor was applied to investigate changes in *A. donax* sap composition to track in vivo and in real-time the plant’s response triggered by the application of severe saline stress conditions. The bioristor confirmed its ability to monitor daily variation of the sensor response (R) in *A. donax* as previously reported in tomato^26^, with R values increasing during the night and rapidly decreasing during the day (Supplementary Fig. 1) both in the apical sensor (AS) and in the basal sensor (BS).

**Figure 2:**
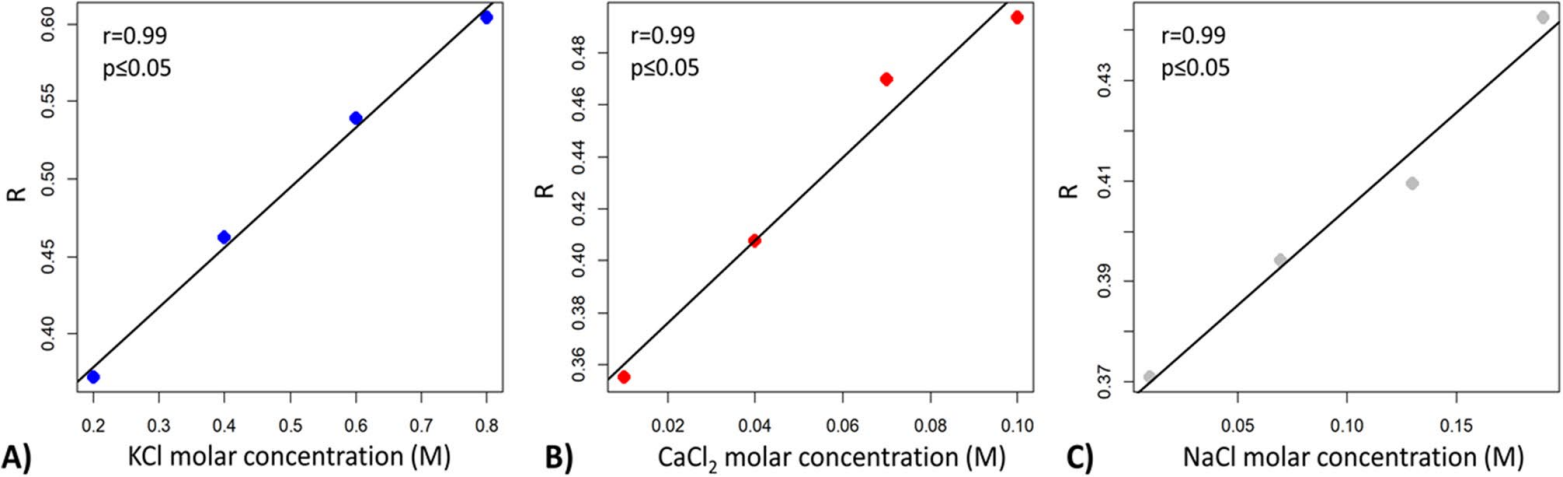
Scatter plots of the correlation between the Sensor Response (R) measured in vivo in the plant sap at 1 V and the molar concentration of (**A**) K^+^ (blue dots); (**B**) Ca^2+^ (red dots); (**C**) Na^+^ (grey dots) measured in the leaves of *A. donax*.

The transfer characteristic of bioristor was verified, in vitro, for Ca^2+^, K^+^, and Na^+^ (Supplementary Fig. 2) using concentrations within the range found in the leaves during the saline stress experiments (see Table 2), demonstrating the possibility to detect through bioristor small changes in the salt related ions concentration. The in vivo test on *A. donax* plants confirmed this property of the bioristor. The linear response of the sensor measured continuously in the plant sap versus the ions concentration measured in Arundo leaves (Fig. 2) showed that the bioristor was able to detect changes in all tested ions.

**Figure 3:**
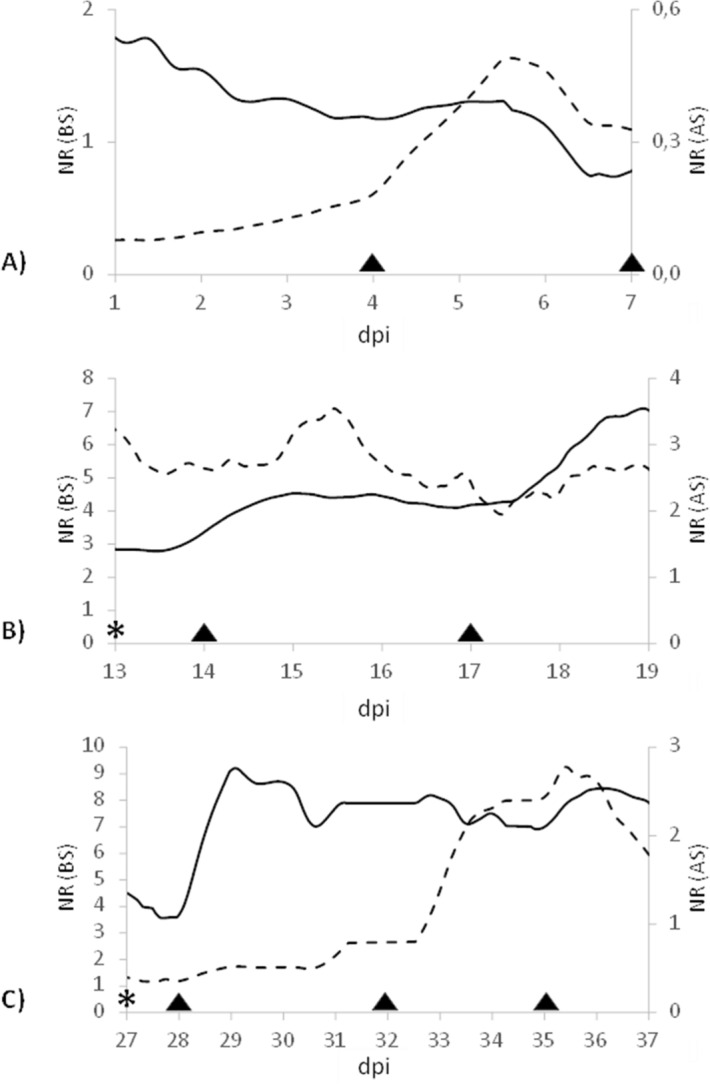
Normalized Sensor Response (NR) of the apical sensor (AS, solid line) and the basal sensor (BS, dashed line) in three different intervals of the experiments: (**A**) initial phase (1–7 days post sensor installation, dpi); (**B**) median phase (13–19 dpi); (**C**) final phase (27–37 dpi). Solid triangles indicate the application of saline treatments. The asterisk indicates the maintenance of the sensor.

**Figure 4:**
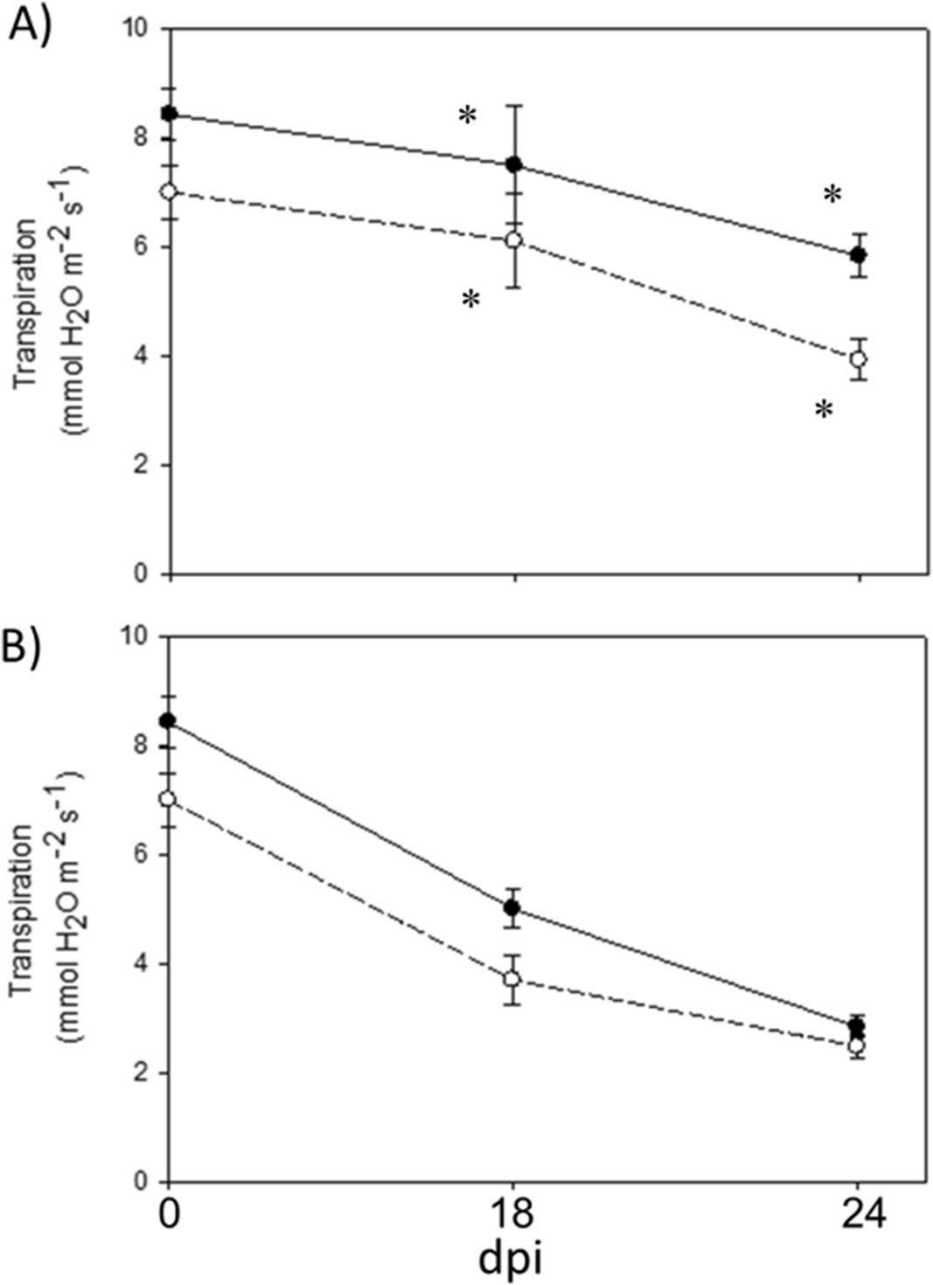
Transpiration rates of control (**A**) and salt stressed (**B**) *A. donax* plants measured at different sampling times: 1, 18 and 24 dpi (see Fig. 3). Black circles indicate measurements collected from the 2nd leaf (apical leaf) and white circles indicate measurements collected form the 5th leaf form the top (basal leaf). Values are means ± standard errors (n = 4). Shapiro–Wilk test was applied to confirm normal distribution of data. Statistically significant differences between means of either apical or basal leaves of control- and salt-stressed plants are indicated by (*) (*P* < 0.05).

To gain more accurate results on the trend of the bioristor response upon saline treatment, the NR (Normalized Response) parameter allowed us to overcome the disturbance due to day/night oscillation. In the initial phase of the experiment (1–7 dpi, days post insertion), the NR of the BS showed a rapid and consistent increase (60%) after addition of Na^+^ (3 dpi, Fig. 3A) up to a maximum recorded at 6 dpi (*p* ≤ 0.001, Fig. 3A, Table 1). Whereas the AS showed a constant and lower response than BS up to 4 dpi (Fig. 3A). In the median phase of the experiment (13–19 dpi; Fig. 3B, Table 1), the NR value of BS significantly increased up to 15.5 dpi (*p* ≤ 0.001), and then decreased up to 17 dpi, suggesting first an ongoing accumulation and compartmentalization of Na^+^ in the basal leaves. Upon a new addition of Na^+^ at dpi 17 (the fifth salt supply, see Fig. 1A), NR of AS showed a new and continuous increase (*p* ≤ 0.001) (Fig. 3B). These data collected in vivo are consistent with the observed general decrease in the transpiration rate measured in salt-stressed plants (Fig. 4).

**Table 1:** Sensor Response (R) of control and salt stressed *A. donax* plants measured at different times: before the beginning of the experiment (0 dpi), in the initial phase (18 dpi), and in the median phase (24 dpi).

dpi		Sensor response (R)		
		Control	Stress	
				p-level (control vs stress)
0	*Apical leaf*	5.24 × 10^−2^ ± 1.72 × 10^−3^	2.81 × 10^−2^ ± 6.03 × 10^−4^	n.s
	*Basal leaf*	3.27 × 10^−2^ ± 1.72 × 10^−3^	8.52 × 10^−3^ ± 6.24 × 10^−4^	n.s
	p-level (apical vs basal)	***	***	
18	*Apical leaf*	2.51 × 10^−2^ ± 1.17 × 10^−3^	4.23 × 10^−2^ ± 9.32 × 10^−4^	***
	*Basal leaf*	1.53 × 10^−2^ ± 7.02 × 10^−4^	8.07 × 10^−2^ ± 1.44 × 10^−3^	***
	p-level (apical vs basal)	***	***	
24	*Apical leaf*	3.94 × 10^−2^ ± 2.70 × 10^−3^	1.03 × 10^−1^ ± 2.56 × 10^−3^	***
	*Basal leaf*	4.52 × 10^−2^ ± 3.51 × 10^−3^	7.72 × 10^−2^ ± 2.1 × 10^−3^	***
	p-level (apical vs basal)	n.s	***	

Values represent the mean of the daily R value ± standard errors (n = 3). ANOVA has been used to assess statistical differences between leaves of control and salt-stressed plants (***p ≤ 0.001; **p ≤ 0.01; *p ≤ 0.05).

The final phase of the experiment covered dpi 27–37. Early in this phase, a stable NR signal was observed in BS. However, NR of BS increased rapidly after the addition of salt (Fig. 1A) at 32 dpi (*p* ≤ 0.001, Fig. 3C, Table 1). On the other hand, an increase of the NR signal was observed in AS at 28 dpi, which was not followed by additional increases after subsequent Na^+^ treatments.

The transpiration rates (TR) measured 18 and 24 dpi in both apical and basal leaves of salt-stressed plants were significantly lower than in control ones (Fig. 4). Na^+^ progressively accumulated in leaves of salt-stressed plants. However, Na^+^ concentration did not show any statistical difference between apical and basal leaves in the median and final phases (Table 2).

**Table 2:** Concentration of Na^+^ (µg g^−1^), K^+^ (µg g^−1^) and Ca^2+^ (µg g^−1^) in control- and salt stressed.

dpi		Na^+^ (µg g^-1^)			K^+^ (µg g^-1^)			Na^+^/K^+^			Ca^2+^(µg g^-1^)		
		Control	Stress		Control	Stress		Control	Stress		Control	Stress	
				*p*-level (control vs stress)			*p*-level (control vs stress)			*p*-level (control vs stress)			*p*-level (control vs stress)
**0**	*Apical leaf*	–	–		49,935.5 ± 3922.2	–		–	–		2562.4 ± 479.8	–	
	*Basal leaf*	–	–		44,195.7 ± 5828.3	–		–	–		4068.9 ± 610.9	–	
	*p*-level (apical vs basal)				ns						*		
**18**	*Apical leaf*	0.0 ± 0.0	7338.4 ± 1124.1	***	34,108.4 ± 789.7	46,975.7 ± 5582.3	*	0.0 ± 0.0	0.48 ± 0.03	***	1581.7 ± 189.8	2770.2 ± 659.7	**
	*Basal leaf*	1070.3 ± 284.4	7033.4 ± 216.6	***	30,336.0 ± 1680.7	33,284.7 ± 6036.5	ns	0.11 ± 0.01	0.66 ± 0.04	**	7418.6 ± .1	6441.9 ± 1199.0	*
	*p*-level (apical vs basal)	***	ns		ns	ns		***	*		***	*	
	*Apical leaf*	0.0 ± 0.0	9024.3 ± 0.5	***	42,085.8 ± 2140.0	37,007.5 ± 0.5	*	0.0 ± 0.0	0.74 ± 0.00	***	2735.7 ± 413.2	6364.3 ± 0.0	***
**24**	*Basal leaf*	3939.4 ± 789.4	8829.3 ± 501.8	***	36,123.7 ± 3347.9	44,650.0 ± 1475.7	*	0.32 ± 0.01	0.59 ± 0.01	**	8957.4 ± 328.9	5438.0 ± 727.8	**
	*p*-level (apical vs basal)	***	ns		ns	ns		***	*		***	*	

*Arundo donax* plants were measured at different sampling times: in the initial phase (0 dpi), in the median phase (18 dpi), and in the final phase (24 dpi). Values represent the mean ± standard errors (n = 4). ANOVA was reported to assess statistical differences between control and salt-stressed plants and between apical and basal leaf level (***p ≤ 0.001; **p ≤ 0.01; *p ≤ 0.05; ns, not significant).

**Figure 5:**
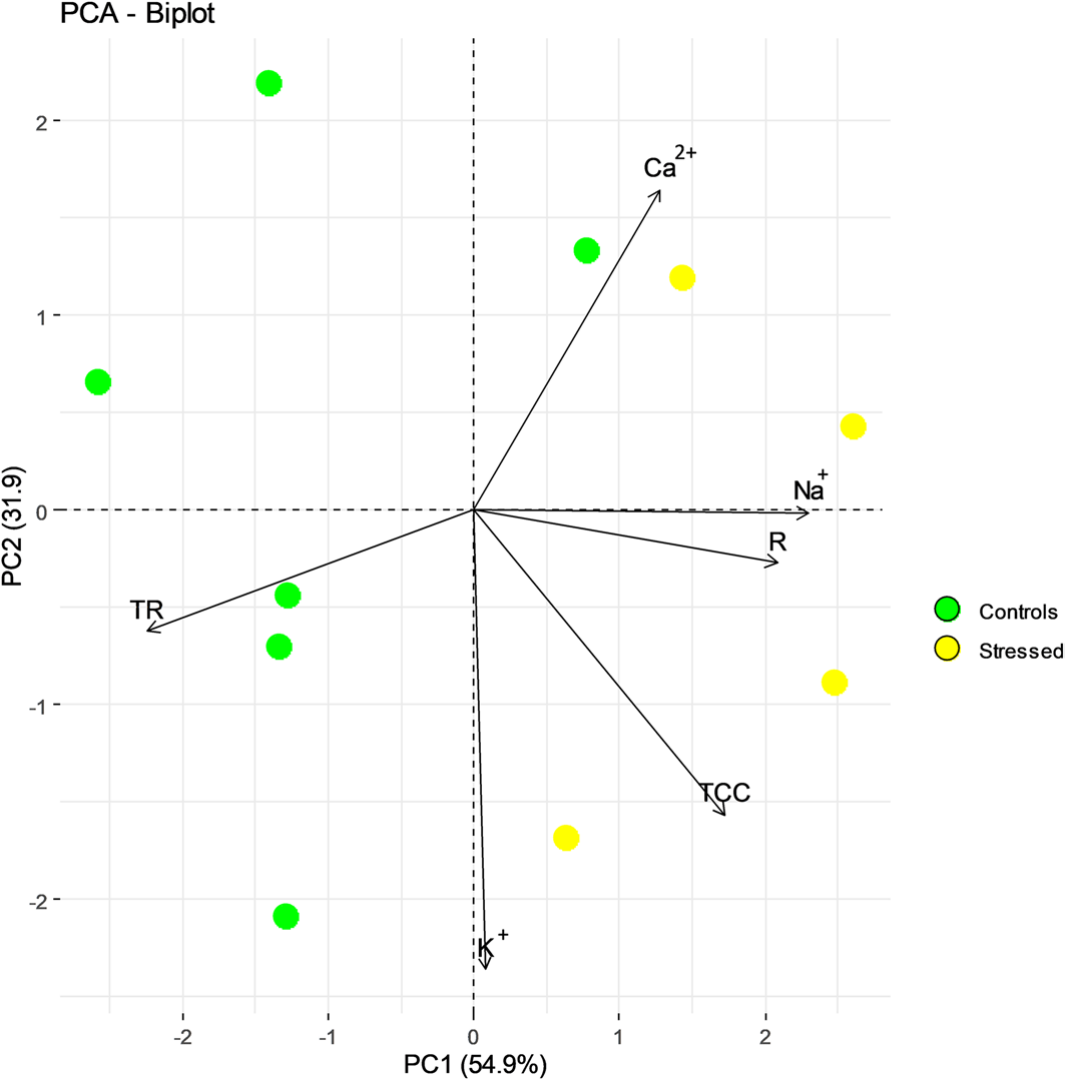
Biplot of the PCA results. The first two PCs display 86.8% of the total physiological variation. The component scores are coloured according to the agronomic groups (yellow dots, stressed plants; green dots control plants). The components loading vectors were proportionally superimposed to their contribution, and their direction indicated the influence of variable group. R, bioristor Response; Na^+^, sodium concentration; K^+^, potassium concentration; Ca^2+^, calcium concentration and TCC, total cations concentration measured in Arundo leaves; TR, transpiration rate.

PCA was performed by considering the measured variables (Na^+^, K^+^ and Ca^+2^ concentration and the total cations concentration and transpiration rate). Of note, to increase the accuracy of the PCA analyses the bioristor’s response R was here considered to allow for the analyses of stressed and control response. Each dot in the diagram (Fig. 5) describes the state of the system measured at specific time points (0, 18, 24 dpi for control plant; 18,24 dpi for stressed plants) for both apical and basal measurement, and the first two components explain 86.8% of the variance (Fig. 5). The first PC (PC1) explained 54.9% of the total variance. In particular, the total cations concentration (TCC) and Na^+^, K^+^ and Ca^+2^ concentrations measured in leaves and R have large positive loading on the PC1 and are positively correlated (Fig. 5). The regularly irrigated controls and the salt-stressed plants are well separated in the biplot indicating the efficacy of the saline treatment.

**Figure 6:**
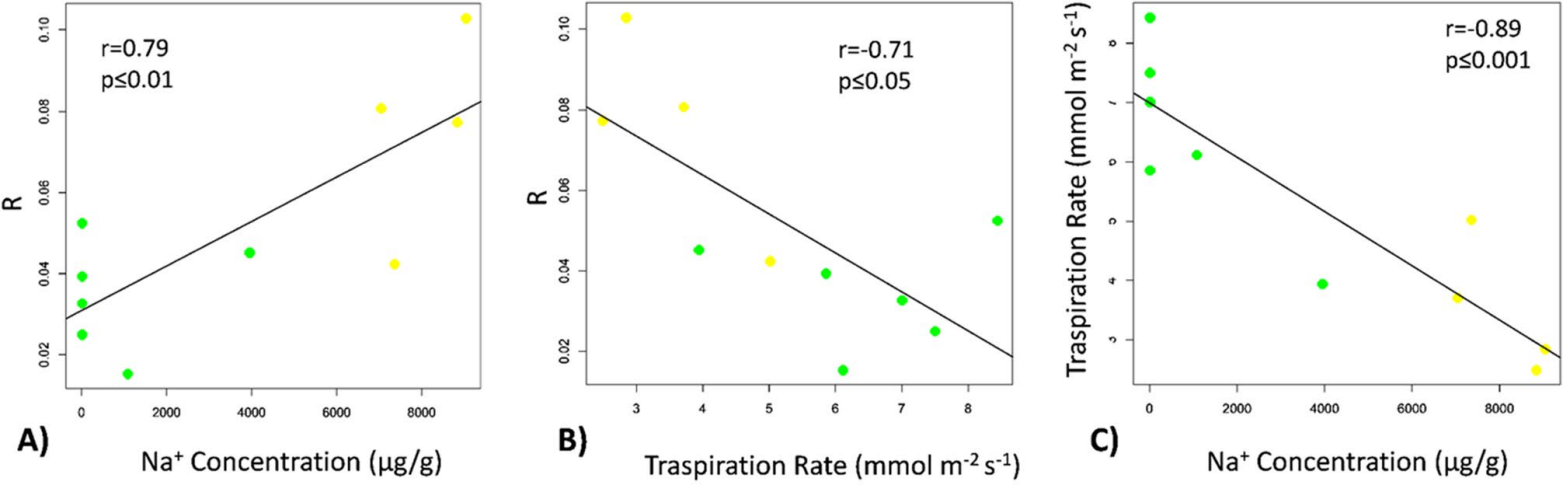
Scatter plots showing the high correlation observed between: the sensor response, R, and Na^+^ concentration measured in leaves (**A**) and transpiration rate, TR, (**B**), and between transpiration rate and Na^+^ concentration (**C**). Green dots indicate the control plants, yellow dots the stressed plants. Pearson correlation coefficient (r) was reported to indicate the statistical significance of observed correlations (*p* ≤ 0.05).

The analyses of the PCA loading vectors showed a positive and high correlation between R and Na^+^ concentration measured in leaves and this is confirmed by the multivariate analysis (Figs. 5,  6A), supporting the efficacy of bioristor in revealing in real-time changes in the plant sap cation concentration during the stress. Furthermore, the analyses of the correlation between R and TR, showed a high and inverse correlation between R and TR (Fig. 6B) that is recognised as strongly affected by the saline stress (Fig. 6C).

The time course analyses of the ions in the plant leaves showed that Na^+^ increased following the salt treatment and the decreased of Ca^2+^ concentration in salt-stressed basal leaves in comparison to the control ones, both at medial and final stages of the salt treatment. Furthermore, K^+^ showed an accumulation in apical leaves only in salt stressed plants in the medial stage, whereas K^+^ decreased in apical leaves and increased in basal leaves at the final stage of stress, compared to respective controls (Table 2, Fig. 2).

## Discussion

This study supports the use of *A. donax* as a valuable energy crop, able to grow in saline soils thus promising for the exploitation of marginal land^10,36^.

Plants have been categorized according to their ability to overcome salt stress: (1) the ability of a plant to survive on saline soils; (2) the absolute plant growth; and (3) the relative growth on saline soil compared to growth on non-saline soils^19,37^.

Here a bioelectronics approach was used to investigate the ability of *A. donax* to growth under adverse growing conditions as high soil salinity exploiting organic chemistry that enables design and tailoring of active materials with desired characteristics, such as functionality, processability, and biocompatibility.

Organic bioelectronics devices and materials have been applied mainly in the context of light-emitting and photovoltaic devices, stretchable and wearable devices, and biomedical applications. However, its usefulness and applicability in plants to monitor and control plant physiology has begun to be explored^26,38–42^. Its use will enhance the possibility to have a continuous monitoring of the physiological processes occurring during the plant growth and upon environmental stress, having a wider picture of the ongoing responses.

Conventionally, punctual detection of the physiological status of plants, a range of technologies and/or their combinations have been applied^43–45^, such as electrical impedance spectroscopy (EIS)^24,25^,chlorophyll fluorescence imaging^46,47^, multispectral imaging^48^, thermal imaging^49^ and electric signals^50^.

Here, an in vivo OECT based sensor, namely bioristor, has been inserted into the stem of the plant. The physiological effects of salt application in *Arundo donax* have been monitored and the ability of bioristor to return a salt stress specific response has been demonstrated. Moreover, being an OECT, the sensor response of the bioristor allows a measurement of the total cation concentration within the plant^30–33^. An increased NR in both BS and AS (Fig. 3) and linear correlation between R and the concentration of cations clearly indicated that the bioristor can effectively reveal presence and changes of all cations in the plant sap. Indeed, R is consistent with the daily transpiration trend due to light-driven stomatal opening and the consequent onset of transpirative passive flux from the soil to the air^51^. Plant growth and development relies on the movement of various ions through the plant sap^52^. The observed circadian variation in sap electrical conductivity of *A. donax* was consistent with the long-distance transport of solutes through the xylem. In fact, ion concentrations are typically higher during the night than in the day^53^ when they are diluted by the higher water transpiration stream^26^.

**Figure 7:**
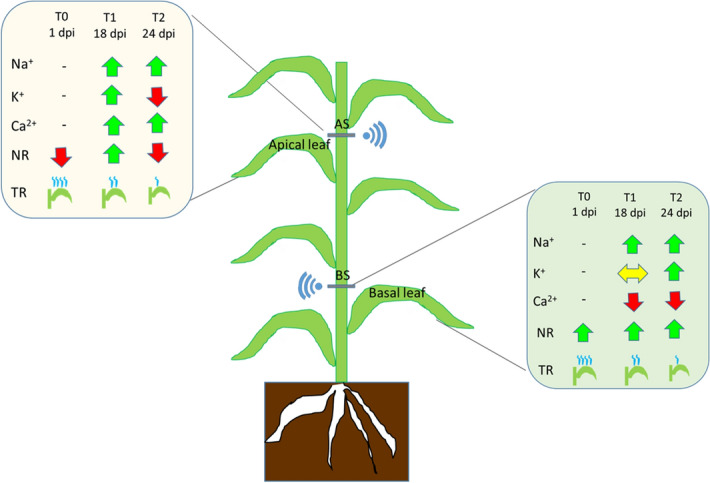
Qualitative visualisation of the bioristor (NR, normalized sensor response); TR, (transpiration rate) and plant ions concentration (Na^+^, sodium; K^+^, potassium; Ca^2+^, calcium) measured in leaves.

Absence of changes in normalized response (NR) values of apical sensor (AS) during the initial stage of the salt treatments (4–6 dpi), may indicate that Na^+^ mainly accumulate in basal leaves of *A. donax* (and it does not reach the apical ones) during the first days upon the treatment (4–6 dpi), despite these data are not supported by quantitative data on ions concentration in leaves. Overall, our results support a positive ion compartmentalization, namely of Na^+^, in the basal portion of the plant during the early phases of the saline stress, following the addition of NaCl to the substrate (Fig. 7). Moreover, the ion transport was described by the NR as follow: the basal sensor (BS) continuously detected a different and higher number of positive ions at lower rate, whereas the upper leaves showed that higher values of ions occurred at a later stage of salinity stress (14 dpi).

When compared with in vivo assessment of leaf transpiration, the bioristor showed a similar trend with the transpiration rate enabling to trace the plant transpiration throughout the measurements of the ions dissolved in the plant sap in the transpiration stream (Fig. 7).

This last result, further support the ability of biostor to detect ions and molecules dissolved and transported through the transpiration stream, suggesting the sensor response as a good proxy of transpiration.

High-resolution sensors are powerful tools to investigate promptly plant responses. In this study case, the bioristor highlighted the potential to synchronize plant functions and environment variations in time series, by providing a continuously monitoring in real-time of the dynamic changes.

The lower transpiration rate (TR) in salt-stressed plants than in control ones and the higher TR in the apical than in basal leaves of salt-stressed plants were expected as root absorption of Na^+^ enhanced the synthesis of Abscisic acid (ABA) that causes a prompt stomatal closure and thus limits transpiration^54,55^. However, the toxic accumulation of Na^+^ and the enhancement of both K^+^ uptake and accumulation imply that Na^+^ and K^+^ transporters and channels play a major role in salt stress tolerance^56^.

The combination of sensors applied in the apical and basal plant parts allowed to hypothesize the trend in time and space of ions or positive electrolytes transport along the culm. The application of the bioristor to *A. donax* plants revealed an initial compartmentalization of the Na^+^, K^+^ and Ca^2+^ ions into the basal leaves during the early stages salt stress, whereas Na^+^ reaches the apical leaves at later stages of the salt stress.

In addition, PCA suggests the sodium uptake as major driver of the variation in the sensor responses between stressed and control plants. This is also supported by the increased Na^+^ concentration measured in treated leaves versus the controls with respect to the other ions measured (Table 2).

The effects of electrical signal in the ion uptake and flow in the plant xylem have been also reported^57,58^ and extensively review in terms of plant physiology and root phenotyping^23,58,59^. Here, we assume as neutral the contribution of the plant sap electronic signal on the bioristor sensor response based on the OECT working principle. This is based on doping-state changes in the semiconductor channel material (PEDOT: PSS) due to electrolyte-ion injections which modify the electrical conductivity^28^. Ions can diffuse through the electrolyte due to electrostatic repulsion and penetrate the polymer matrix thanks to the application of a gate voltage selectively applied (Vg) that controls the infiltration of ions into the channel and, consequently, the material’s doping extent/degree (redox state). The extent of the reduction of the channel current is measured in the time window relative to the application of the gate voltage (6 min) and is, thus, strictly dependent on the ions concentration in the electrolyte solution in the OECT geometry (0,5–1 cm between the gate and the channel of the OECT). To support this hypothesis, we observed that if the voltage applied to the gate is 0 V, the sensor response R is also 0, excluding any interference of electrophysiology signals in the sensor response.

The ongoing development and application of a bioristor functionalized with ion-selective membranes, will further help in deciphering the mechanisms occurring in the ion movements in saline stress conditions^60^.

The application of the bioristor on several plant species and its development for the ion specific detection can significantly improve in vivo plant phenotyping and the real-time monitoring of salt stress for the identification of salt resilient genotypes.

The high sensitivity of bioristor and in general of OECT sensors towards physiological changes occurring in the plant sap, supports the effectiveness of bioristor as tool for in vivo phenotyping studies and precision agriculture to bridge electronics and biological systems.

## Methods

### Plant material and salt (NaCl) stress application

*A. donax* plants were propagated by rhizomes collected in Sesto Fiorentino (43°81′75″ N, 11°18′88″ E) (Italy). Rhizomes were kept in tap water for one day before to be planted in 6 L pots containing quartz sand (substrate). Plants were obtained from rhizomes in two months and then kept growing in a climatic chamber under controlled environmental conditions: maximum and minimum (day/night) temperature of 30 °C and 22 °C, respectively; maximum and minimum relative air humidity of 60% and 40%, respectively; photosynthetic photon flux density (PPFD) of 700 μmol m^−2^ s^−1^ for 14 h per day. Plants were watered weekly with full strength Hoagland solution in order to supply mineral nutrients to optimal level until the beginning of the experiment.

The experiment was performed by growing two different groups of 10 *A. donax* plants: (a) under optimal nutritional conditions by applying the standard Hoagland solution (control); (b) by applying Hoagland solution where 200 mM NaCl (Na^+^ salt stress) were added. These solutions were supplied twice a week; the treatment lasted 37 days (Fig. 1A).

### Bioristor: sensor description, data acquisition and analysis

The bioristor is an Organic Electrochemical Transistor (OECT) whose channel and gate electrodes, both constituted by a textile fiber functionalized with a conductive polymer^31^, were directly integrated into the plant stem (Fig. 1B). For the functionalization, two textile fibers were soaked for 5 min in aqueous poly(3,4-ethylenedioxythiophene) doped with polystyrene sulfonate (CleviosPH500, Starck GmbH, Munich, Germany), after which ethylene glycol (10% v/v) and dodecyl benzene sulfonic acid (2% v/v) were added. The fibers were then baked at 130 °C for 90 min. Before functionalization, each thread was cleaned with a plasma oxygen cleaner treatment (Femto, Diener electronic, Ebhausen/Germany) to increase its wettability and facilitate the adhesion of the aqueous conductive polymer solution. A treated fiber was inserted into the stem of each *A. donax* plant, until it passed to the opposite side of the stem. The fiber was connected on each end to a metal wire with silver paste to stabilize the connections, forming the “source” and “drain” electrodes. The transistor device was completed by introducing a second functionalized textile thread as the gate electrode to reduce the drawback effects caused by the silver wire used for tomato plants^26^. The bioristor elements were connected to a NI USB-6343 multifunction I/O device (National Instruments, Austin, TX, USA) (Fig. 1).

The bioristor drain and gate current play a major role in determining the sensor response. The p-type doped PEDOT (oxidized from the electrochemistry point of view) leads to mobile holes generating a hole current (I_ds0_) which flows in the channel when a drain voltage (V_ds_ = − 0.05 V) is applied. These holes are balanced by the negative charge of the PSS sulfonate group^61^, until the application of a positive gate bias (V_gs_ = 0.6 V), which leads to the injection of cations (M^+^) from the electrolyte (xylem sap in this case) into the PEDOT: PSS channel causing its de-doping, according to equation^62^:

1 $${\text{PEDOT}}^{ + }: {\text{PSS}}^{ - } + {\text{ M}}^{ + } + {\text{e}}^{ - } \, \to {\text{PEDOT}}^{0} + {\text{ M}}^{ + }: {\text{PSS}}^{-}$$

The “de-doping process”^63^, according to the reduction of the oxidized PEDOT^+^ to PEDOT^0^ and the decrease of the number of holes in the channel, leads to a drop in the drain current (I_ds_). The whole process is reversible: when gate-source voltage is switched off (V_gs_ = 0 V), cations diffuse from the channel to the electrolyte increasing the number of conducting holes and, consequently reduced PEDOT^0^ returns to the oxidized state and drain-source current to the initial value (I_ds0_). During the entire experiment, the 0.6 V gate-source voltage is turned on for 6 min and then it is turned off; the measurement is repeated every 24 min.

The sensor response (R), obtained every 24 min, can be expressed as

2 $${\text{R }} = \, \left| {{\text{I}}_{{{\text{dS}}}} - {\text{ I}}_{{{\text{dS}}0}} } \right|/{\text{I}}_{{{\text{dS}}0}}$$

where Ids is the value of the drain current just before the gate-source voltage is turned off. R is related to the cation concentration in the electrolyte solution^22^, thus allowing the monitoring of the temporal variation in the plant sap’s cationic content. Here, R measured in stressed plants (R_stress_) and in control plants (R_control_) is reported.

When monitoring the plant sap concentration over several days it showed to be useful to smooth out the day/night signal oscillations due to plant circadian rhythms that characterize the bioristor response^19^. Thus, the ratio between the signal recorded form sensors installed in salt-stressed and control plants was expressed as Normalized Response (NR);

3 $${\text{NR}} = {\text{R}}_{{{\text{stress}}}} /{\text{R}}_{{{\text{control}}}}$$

To investigate the possibility to track the ion transport (in specific of the Na^+^ ion) along the plant culms of *A. donax* plants, two bioristor sensors were integrated at two different heights along the same culm, the apical sensor (AS) between the 2nd and 3rd leaf and the basal sensor (BS) between the 5th and 6th leaf from the apex (Fig. 1C) in 3 control and 3 salt-stressed *A. donax* plants (for a total of 12 sensors). From the overall data acquired (37 days), three intervals, defined from the day of installation (dpi; 1–7 dpi; 13–19 dpi; 27–37 dpi) have been selected as the most representative of the entire set of measurements due to both their low background noise and accuracy which have been indicated as initial (1–7 dpi), median (13–19 dpi) and final (27–32 dpi) phase. The median and final phases began with a sensor maintenance causing a variation in the recorded values due to the new sensor’s part integration. Therefore, these three intervals should be read as independent measurements and therefore the moving average of the NR was evaluated for each of those.

The operability of the bioristor was validated within the range of sodium (Na^+^), calcium (Ca^2+^) and potassium (K^+^) concentrations detected in the leaves; the sensor response was measured in vitro by using different solutions at low concentrations of Na^+^ (0.01 to 0.19 M), K^+^ (0.2 to 0.8 M), and Ca^2+^ (0.01–0.1 M). Moreover, the sensor response was evaluated as V_ds_ = − 0.05 V and V_gs_ in the 0–1 V voltage range using 0.2 V steps. Transfer characteristics are expressed as Sensor Response (R) vs V_gs_.

### Plant physiological response to salinity

The transpiration flux of H_2_O (TR) was measured in vivo in the 2nd and 5th leaf (from the shoot apex) of *A. donax* plants by using a portable gas exchange system (Li-Cor 6400, Li-Cor Biosciences Inc., NE, USA). A portion of leaf was clamped in the 2 cm^2^ Li-Cor cuvette and exposed to a constant PPFD of 1000 μmol m^−2^ s^−1^, CO_2_ concentration of 400 ppm, temperature of 30 °C and relative humidity (RH) ranging between 45 and 50%. After reaching steady-state conditions, TR and PR were calculated according to the formulations of von Caemmerer et al.^64^. Analysis was performed in leaves sampled at different days post insertion (dpi) of the sensor: 1 dpi, 14 dpi, 24 dpi.

The concentrations of Na^+^, Ca^2+^ and K^+^ (µg g^−1^ DW) accumulated in *A. donax* plants were determined in 1 g of leaves by flame atomic absorption spectrometry (Analyst 200, Perkin Elmer). Analysis of these mineral elements was performed in the same leaves following gas exchange measurements.

### Statistical analyses

All data retrieved from the sensors were statistically analysed by applying a multi-comparison approach using the Analysis of Variance (ANOVA) in MatLab 2014a (8.3.0.532). Principal components analyses (PCA) was performed using the R “prcomp” function and represented as a biplot by using the R package factoextra^65,66^. The first two principal components PC1 and PC2 and the corresponding component loading vectors were visualized and summarized in a biplot, in which component scores (indicated in dots) are coloured according to thesis classification. Analysis of variance (ANOVA) was performed to assess the effect of Na^+^ supply in *A. donax* plants (*p* ≤ 0.05 level).

## Supplementary Information

Supplementary Information.

## Data Availability

The data supporting the findings of this study are available from the corresponding authors upon request.
