# Supplementary material for: Real-time monitoring of Arundo donax response to saline stress through the application of in vivo sensing technology
Source: Sci Rep. 2021 Sep 20;11:18598. doi: 10.1038/s41598-021-97872-6 (PMC8452760; doi:10.1038/s41598-021-97872-6)
Supplement: Supplementary file 1 — Supplementary Information. [file 41598_2021_97872_MOESM1_ESM.docx]

**Real-time monitoring of *Arundo donax* response to saline stress through the application of *in vivo* sensing technology**

Janni Michela^1,2*^, Cocozza Claudia^3*^, Brilli Federico^4^, Pignattelli Sara^4^, Vurro Filippo^1^, Coppede Nicola^1^, Bettelli Manuele^1^, Calestani Davide^1^, Francesco Loreto^5^, Andrea Zappettini^1^

^1^ National Research Council of Italy, Institute of Materials for Electronics and Magnetism (IMEM), National Research Council (CNR), Parco Area delle Scienze 37/A, 43124 Parma

^2^ National Research Council of Italy, Institute of Bioscience and Bioresources (IBBR), National Research Council (CNR), Via Amendola 165/A, 70126 Bari, Italy

^3^ University of Florence, Department of Agriculture, Food, Environment and Forestry (DAGRI), 50145 Florence, Italy

^4^ National Research Council of Italy, Institute for the Sustainable Plant Protection (CNR - IPSP), Via Madonna del Piano 10, 50019 Sesto Fiorentino, Italy

^5^ National Research Council of Italy – Department of Biology, Agriculture and Food Sciences (CNR-DISBA), P. le Aldo Moro, 00185 Roma, Italy

* corresponding author: [michela.janni@imem.cnr.it](mailto:michela.janni@imem.cnr.it)

* corresponding author: claudia.cocozza@unifi.it

**Supplementary Material**


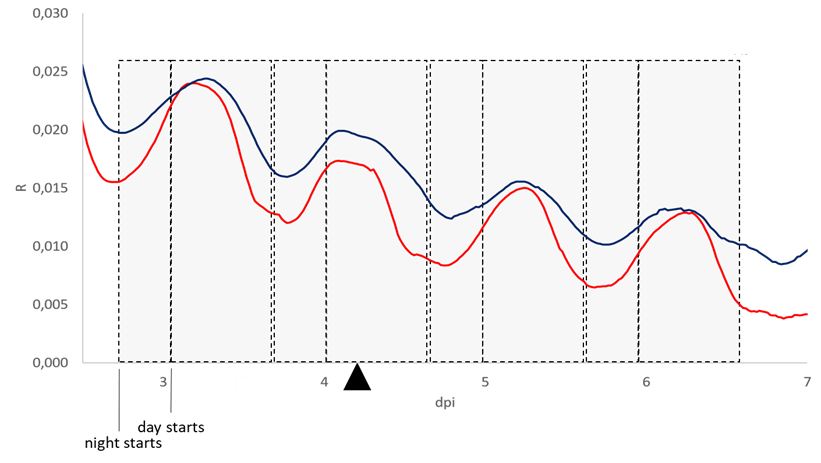


**Supplementary Fig 1.** Sensor response (R) calculated as average over a period of 5 days (3-7 dpi). Grey boxes refer to a day period. Triangle indicates the application of saline treatment. Each line is the mean of data collected in 3 plants.


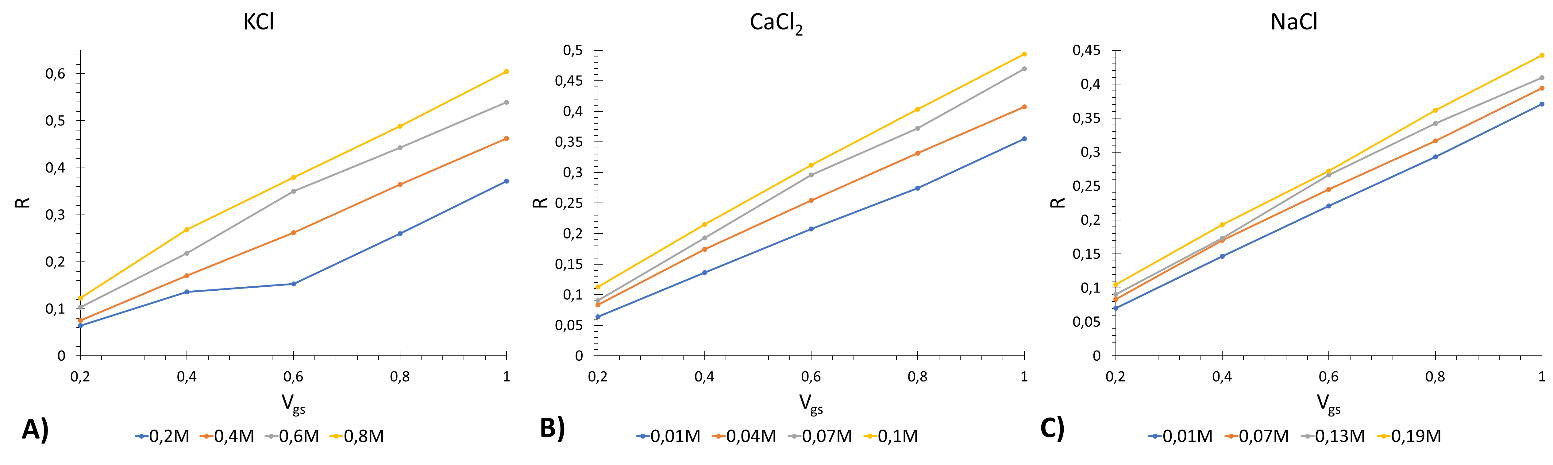


**Supplementary Figure 2**. Transfer characteristics of the Sensor Response (R) measured using different concentration of A) K^+^, B) Ca^2+^ and C) Na^+^ salts, as a function of gate voltage from 0.2 to 1 V with 0.2 V step.
